# Dysplasie fibreuse osseuse crânio-faciale: à propos de six observations

**DOI:** 10.11604/pamj.2020.37.271.21350

**Published:** 2020-11-25

**Authors:** Cheikh Ahmedou Lame, Birame Loum, Thierno Boubacar Diallo, Cheikhna Ba Ndiaye, Khady Marie Agnès Diouf, Amat Fall

**Affiliations:** 1 Service d´ Otorhinolaryngologie (ORL) et de Chirurgie Cervico-Faciale, Hôpital Principal de Dakar, Dakar, Sénégal,; 2 Département d´Imagerie Médicale, Hôpital Principal de Dakar, Dakar, Sénégal

**Keywords:** Dysplasie fibreuse osseuse, dysplasie fibreuse crânio-faciale, tumeur osseuse, syndrome de McCune-Albright, Bone fibrous dysplasia, craniofacial fibrous dysplasia, bone tumor, McCune-Albright´s syndrome

## Abstract

La dysplasie fibreuse osseuse (DFO) est une maladie osseuse bénigne, congénitale et rare, dans laquelle l´os normal est remplacé par du tissu fibro-osseux entraînant des lésions osseuses déformantes. Tous les os peuvent être concernés, cependant la localisation crânio-faciale se particularise par ses manifestations cliniques, son évolution et ses difficultés thérapeutiques. L´objectif de notre travail était de décrire les aspects diagnostiques, thérapeutiques et évolutifs des DFO crânio-faciales. Nous rapportons six observations de patients suivis au service d´ORL de l´Hôpital Principal de Dakar pour DFO crânio-faciale. L´âge moyen des patients était de 26,16 ans avec des extrêmes de 11 et 58 ans. Le sexe féminin était prédominant dans 83% des cas. Les circonstances de découverte étaient dominées par la déformation osseuse. Un patient a consulté pour obstruction nasale unilatérale avec épistaxis. Le scanner a permis dans tous les cas de faire le diagnostic et le bilan topographique. Deux patientes ont bénéficié d´un traitement chirurgical. Un cas de récidive a été observé. La DFO crânio-faciale est une pathologie osseuse rare qui peut se manifester par de graves troubles sensoriels et fonctionnels. Elle pose de réelles difficultés thérapeutiques et fait appel à une bonne collaboration interdisciplinaire.

## Abstract (en)

Fibrous dysplasia (FD) of bone is a benign, congenital and rare disease in which normal bone is replaced by fibrous bone tissue, resulting in bone deformities. It can affect any bone in the body, however craniofacial fibrous dysplasia is characterized by specific clinical manifestations, progression and therapeutic issues. The purpose of our study was to describe the diagnostic, therapeutic and evolutionary features of craniofacial FD. This study involved six patients with craniofacial FD followed up in the Department of Otolaryngology at the Principal Hospital of Dakar. The average age of patients was 26.16 years, ranging from 11 to 58 years. Sex ratio favoured women (83% of the cases). Bone deformity was the main feature of craniofacial FD leading to diagnosis. One patient presented with unilateral nasal obstruction with epistaxis. In all cases, scanner enabled diagnosis and topographic balance. Two female patients underwent surgery. One case of recurrence was reported. Craniofacial FD is a rare bone disease that can manifest as serious sensory and functional disorders. It poses real therapeutic issues; hence adequate interdisciplinar management is essential.

## Introduction

La dysplasie fibreuse osseuse (DFO) est une anomalie rare du développement du squelette, dans laquelle un os normal est remplacé par une prolifération excessive de tissu conjonctif fibreux mélangé à des trabécules osseuses irrégulières. Il peut s'agir d'une lésion unique appelée monostotique ou de lésions multiples qui affectent de nombreux os, c´est la forme polyostotique [1-3]. La localisation crânio-faciale est rare. Les auteurs rapportent six observations de patients ayant présenté une DFO crânio-faciale.

## Méthodes

Il s´agissait d´une étude rétrospective descriptive menée au service d´ORL de l´Hôpital Principal de Dakar (Sénégal) entre janvier 2011 et décembre 2019. Elle a porté sur tous les patients suivis pour DFO crânio-faciale. Les paramètres étudiés étaient l´âge, le sexe, les antécédents des patients, la présentation clinique, les données paracliniques, le traitement proposé et l´évolution de la maladie.

## Résultats

**Figure 1:**
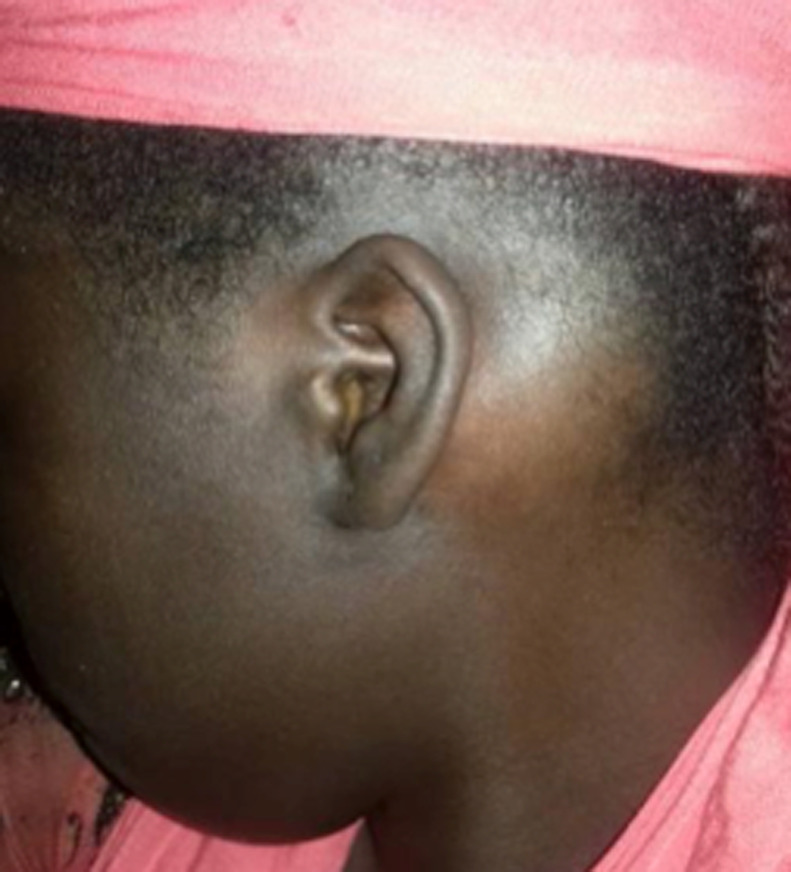
patiente N^o^ 4 présentant une tuméfaction osseuse mastoïdienne gauche

**Figure 2:**
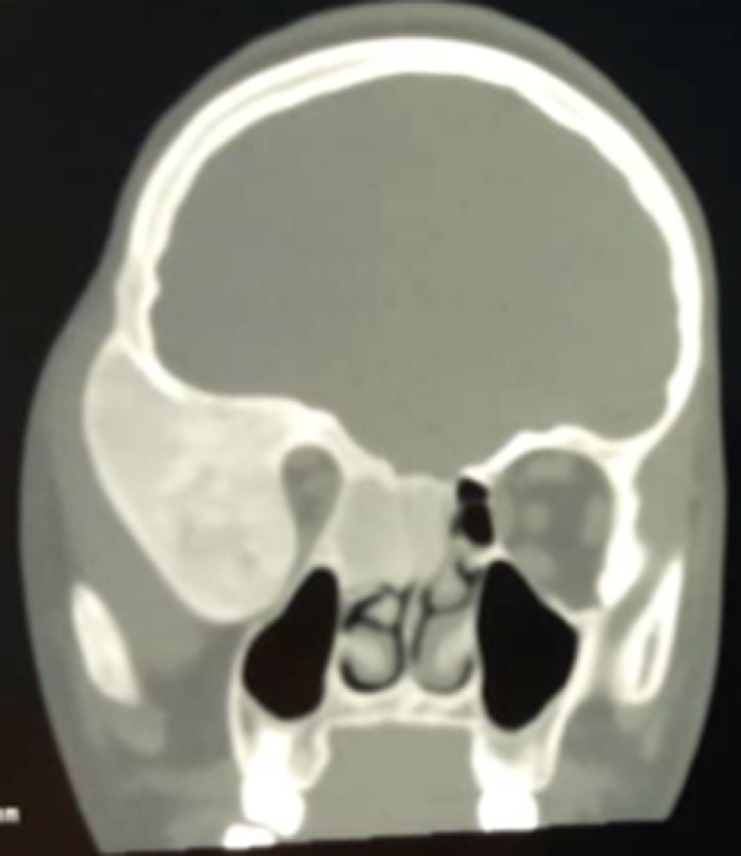
tomodensitométrie (TDM) en coupe coronale montrant un processus tumoral osseux avec aspect de verre poli développé aux dépens de l’os temporal et sphénoïdal droits

**Figure 3:**
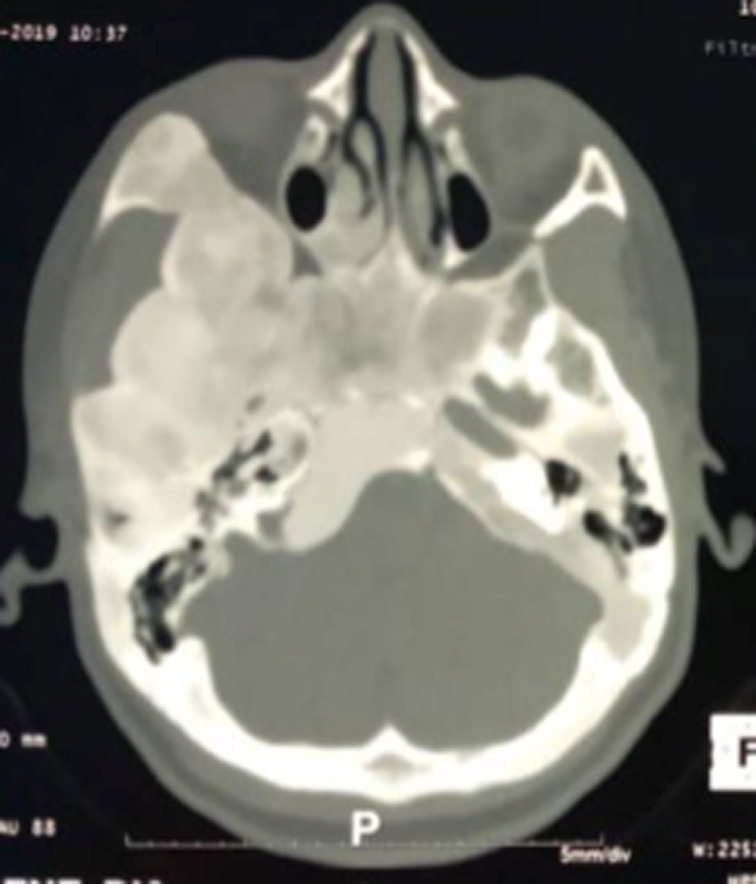
TDM en coupe axiale montrant un processus tumoral osseux avec aspect de verre poli développé aux dépens de l’os temporal et sphénoïdal droits

**Figure 4:**
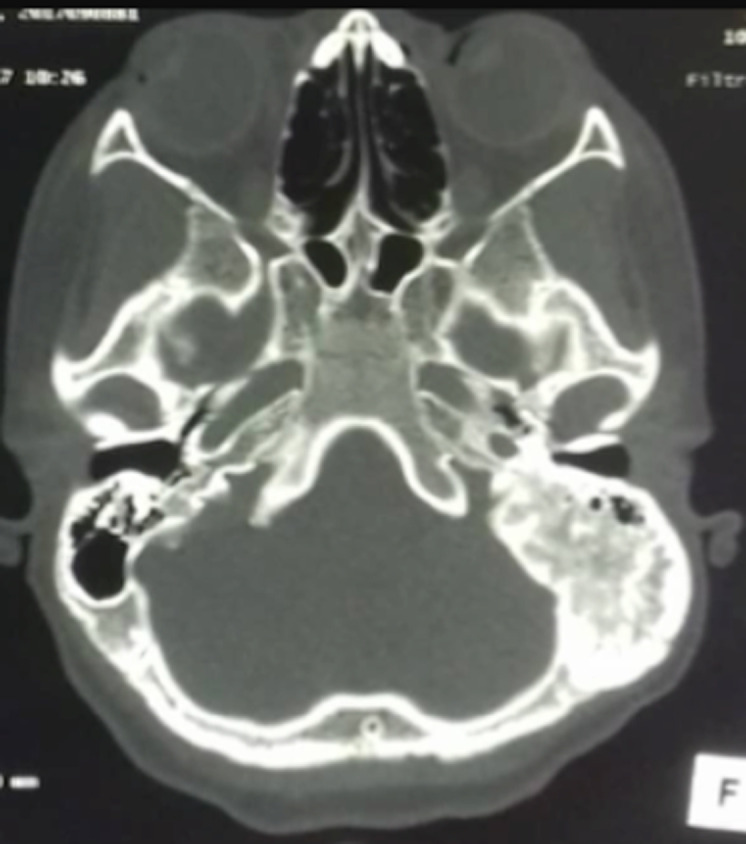
TDM en coupe axiale montrant un processus tumoral osseux avec aspect de verre poli développé aux dépens de la mastoïde

Six patients ont été inclus: 5 femmes et un homme. L´âge moyen était de 26,16 ans avec des extrêmes allant de 11 à 58 ans. Une patiente avait dans ses antécédents deux gestes de chirurgie maxillo-faciale. Un patient avait des antécédents de sinusite associée à des épistaxis à répétition. Cinq patients avaient consulté pour une tuméfaction du crâne ou de la face (Figure 1), douloureuse chez un seul malade. Un patient s´était présenté pour une obstruction nasale chronique gauche associée à une épistaxis homolatérale. Un nodule thyroïdien était retrouvé chez une patiente avec des signes de puberté précoce sans lésion dermatologique objectivée avec une euthyroïdie biologique. Aucun patient n´a présenté de troubles visuels (Tableau 1). Le scanner du massif facial montrait chez tous les patients une prolifération osseuse avec un aspect de verre dépoli, évocateur de dysplasie fibreuse osseuse. La lésion concernait un seul os, le maxillaire chez 3 patients, la base du crâne chez une (Figure 2 et Figure 3), la mastoïde chez une autre (Figure 4) et l´os frontal chez une dernière. Un patient a bénéficié d´un traitement antalgique à base d´AINS qui a permis de contrôler la douleur. Deux patientes ont bénéficié d´une hémi-maxillectomie. Trois autres sont sous surveillance sans aucun traitement. L´évolution était marquée par le rétrécissement du méat auditif externe chez une jeune patiente de 11 ans, chez qui la décision de retarder la chirurgie a été retenue avec la famille. Une patiente ayant bénéficié d´une hémi-maxillectomie 8 ans auparavant présente une récidive locale sur moignon évoluant très lentement. Tous les patients sont régulièrement suivis.

**Tableau 1:** données cliniques, paracliniques et thérapeutiques

Patient	Age (ans)	Sexe	Antécédents	Présentation clinique	Biologie	Biopsie osseuse	Traitement	Evolution
1	48	F	2 chirurgies maxillaires	Tuméfaction dure de la joue droite	Pas de bilan	Non	Hémi-maxillectomie	Récidive locale
2	11	F	Néant	Tuméfaction temporale droite non douloureuse, nodule thyroïdien droit, puberté précoce, rétrécissement du MAE	Bilan thyroïdien normal, bilan phosphocalcique normal	Non	Surveillance	Suivi régulier ORL, ophtalmologique et pédiatrique
3	12	F	Néant	Tuméfaction douloureuse frontale depuis un an sans troubles visuels	Pas de bilan	Non	Surveillance	Suivi régulier
4	12	F	Néant	Tuméfaction mastoïdienne gauche chronique sans signe cochléo-vestibulaire ni déficit moteur facial	Non	Non	Néant	Surveillance régulière
5	58	M	Sinusite	Obstruction nasale et épistaxis gauches	Non	Néant	Corticoïde nasal	Disparition des signes rhinologiques, surveillance
6	16	F	Néant	Tuméfaction joue	Non	Non	Hémi-maxillectomie	Suivi régulier

## Discussion

La DFO, est un hamartome dans lequel l´os normal est remplacé par du tissu fibreux avec métaplasie osseuse secondaire par défaut de maturation des ostéoblastes, aboutissant ainsi à un os nouvellement formé et peu calcifié [1,4-7]. Elle est due à une mutation du gène GNAS-1 situé sur le chromosome 20q13 et qui code pour une sous-unité alpha de la protéine G [4-6,8]. Tous les os de l´organisme peuvent être atteints. L´affection peut toucher un seul os, c´est la forme monostique; ou s´étendre à plusieurs os, c´est la forme polyostotique [1,4,5,8]. La localisation crânio-faciale est relativement rare et représente 10 à 20% de tous les sites [1,3,4]. C´est essentiellement une affection de l´enfant et de l´adulte jeune [1] comme le montre notre série, sans prédilection pour un sexe selon différents auteurs [5,8]. Dans notre série, la prédominance féminine est nette. L´explication pourrait être un souci esthétique plus marqué chez la femme dans nos sociétés. Sur le plan clinique, la DFO peut être asymptomatique ou se manifester par des tuméfactions osseuses déformantes, des douleurs osseuses ou des fractures pathologiques [1,3,4,8,9] . La localisation crânio-faciale (mandibule, maxillaire, voûte, base du crâne) se particularise par une symptomatologie spécifique pouvant révéler la maladie. Ainsi, l'atteinte des os frontal, sphénoïdal, nasoéthmoïdal et maxillaire peut entraîner un syndrome rhino-sinusien [1] comme chez notre patient N^o^ 5. Des signes sensitifs à type de dysesthésies faciales, de céphalées, ou des troubles sensoriels ophtalmologiques ou cochléo-vestibulaires peuvent émailler l´évolution de la maladie [1,5,7,8]. La DFO peut s´associer à des manifestations extra-squelettiques (taches cutanées « café au lait », puberté précoce, endocrinopathies sécrétantes) pour définir le syndrome de McCune-Albright trouvé chez une de nos malades (patiente 2). La combinaison entre DFO et myxomes intramusculaires correspond au syndrome de Mazabraud [4,5,8,9]. Dans la DFO, la radiographie standard montre un aspect radio-transparent voilé ou en verre dépoli des os atteints [1,2].

La scintigraphie osseuse est généralement recommandée pour exclure la variante polyostotique de la maladie ou en cas de lésion atypique ou suspecte de malignité [1,8]. La tomodensitométrie objective des images caractéristiques décrites comme du verre dépoli dans 56%, homogène dense (sclérotique) dans 23% et radiotransparent (kystique) dans 21% [1]. Elle permet de faire la topographie des os atteints et de suivre l´extension [1,2,8]. L´imagerie par résonance magnétique (IRM) est surtout importante pour évaluer l´atteinte des nerfs crâniens. Le scanner couplé à l´IRM permet de définir l´effet compressif de la DFO crânio-faciale sur l´orbite, les canaux optiques et les zones adjacentes (sinus paranasaux) [1]. La biopsie osseuse n´est pas systématique dans la DFO crânio-faciale [5]. Aucun de nos patients n´en a bénéficié, en préopératoire. L´étude histologique de la lésion osseuse suspecte n´est souvent nécessaire qu´en cas de présentation inhabituelle et/ou de suspicion de malignité [2]. Elle confirme le diagnostic en mettant en évidence un stroma fibreux modérément cellulaire entourant des trabécules curvilignes irrégulières d'os tissé disposé en lettres chinoises avec présence d'ostéocytes et sans aucun anneau ostéoblastique [2,7]. Il n´existe pas de traitement spécifique de la DFO. Un traitement antalgique classique (paracétamol, AINS, opioïdes) sera proposé en cas de douleurs osseuses. Les bisphosphonates seront utilisés dans les douleurs persistantes modérées à sévères (EVA > 3/10) [2]. Leur efficacité à augmenter la densité osseuse locale et à prévenir les complications n´est pas démontrée [1,2]. La chirurgie est indiquée dans des cas précis de DFO crânio-faciale (maladie symptomatique, en cas de troubles neurosensoriels ou à visée esthétique) [2,6,8,10]. Elle sera partielle voire radicale, suivie d´une reconstruction dans les localisations qui le permettent (voûte crânienne, os frontal, maxillaire, mandibule) [2]; ailleurs, elle sera décompressive en cas d´atteinte de certains organes nobles de la base du crâne (nerf optique, ganglion trigéminal, orbite, conduit auditif externe) [2]. Deux de nos patients ont bénéficié d´une chirurgie maxillaire à visée esthétique. L´étude histopathologique des pièces opératoires a permis de confirmer le diagnostic de DFO. La récidive est la règle en cas de chirurgie conservatrice (comme chez la patiente N^o^ 1). La transformation maligne sarcomateuse est possible dans 1% des cas, surtout dans la forme polyostotique [3,4]. Dans tous les cas, la surveillance doit être régulière, prolongée, multidisciplinaire impliquant ophtalmologistes, ORL, orthopédistes, neurochirurgiens, endocrinologues et psychologues [7,8,10].

## Conclusion

La DFO crânio-faciale est une pathologie osseuse rare qui peut se manifester par des troubles esthétiques et fonctionnels importants. Elle pose, dans certains cas, de réelles difficultés de prise en charge et fait appel à une bonne collaboration interdisciplinaire dans le cadre d´un suivi continu des patients, même après chirurgie.

### Etat des connaissances sur le sujet

- La dysplasie fibreuse osseuse est une maladie rare;
- La localisation crânio-faciale est exceptionnelle;
- Le traitement de cette forme est très difficile.

### Contribution de notre étude à la connaissance

- Très peu d´études ont été réalisées en Afrique;
- Notre série souligne toutes les difficultés liées à la prise en charge de cette pathologie dans notre contexte d´exercice.
